# Crystal structure of glutamate-1-semialdehyde-2,1-aminomutase from *Arabidopsis thaliana*

**DOI:** 10.1107/S2053230X16007263

**Published:** 2016-05-23

**Authors:** Yingxian Song, Hua Pu, Tian Jiang, Lixin Zhang, Min Ouyang

**Affiliations:** a Photosynthesis Research Center, Key Laboratory of Photobiology, Institute of Botany, Chinese Academy of Sciences, Beijing 100093, People’s Republic of China

**Keywords:** X-ray crystallography, asymmetry, pyridoxamine 5′-phosphate, pyridoxal 5′-phosphate, gating loop, glutamate-1-semialdehyde-2,1-aminomutase, *Arabidopsis thaliana*

## Abstract

Glutamate-1-semialdehyde-2,1-aminomutase (GSAM) catalyzes the isomerization of glutamate-1-semialdehyde (GSA) to 5-aminolevulinate (ALA) and is distributed in archaea, most bacteria and plants. Although structures of GSAM from archaea and bacteria have been resolved, a GSAM structure from a higher plant is not available, preventing further structure–function analysis. Here, the structure of GSAM from *Arabidopsis thaliana* (*At*GSA1) obtained by X-ray crystallography is reported at 1.25 Å resolution. *At*GSA1 forms an asymmetric dimer and displays asymmetry in cofactor binding as well as in the gating-loop orientation, which is consistent with previously reported *Synechococcus* GSAM structures. While one monomer binds PMP with the gating loop fixed in the open state, the other monomer binds either PMP or PLP and the gating loop is ready to close. The data also reveal the mobility of residues Gly163, Ser164 and Gly165, which are important for reorientation of the gating loop. Furthermore, the asymmetry of the *At*GSA1 structure supports the previously proposed negative cooperativity between monomers of GSAM.

## Abstract 2

A structural study of *A. thaliana* glutamate-1-semialdehyde-2,1-aminomutase (GSAM) has revealed asymmetry in cofactor binding as well as in the gating-loop orientation, which supports the previously proposed negative cooperativity between monomers of GSAM.

## 1. Introduction   

Tetrapyrroles such as chlorophyll and haem are cofactors that are essential for a wide variety of crucial biological processes, including photosynthesis and respiration (Mochizuki *et al.*, 2010 ▸). 5-Aminolevulinic acid (ALA) is the universal precursor of tetrapyrroles (Porra, 1997 ▸; Reinbothe & Reinbothe, 1996 ▸; von Wettstein *et al.*, 1995 ▸). Plants, green algae and the majority of bacteria synthesize ALA through the C5 pathway using tRNA-bound glutamate as a substrate (Ilag & Jahn, 1992 ▸; Jahn *et al.*, 1991 ▸, 1992 ▸; Kannangara & Gough, 1978 ▸; Kannangara *et al.*, 1988 ▸). The activated glutamate is first reduced to glutamate-1-semialdehyde (GSA) by the NADPH-dependent glutamyl-tRNA reductase (GluTR; EC 1.2.1.70; Moser *et al.*, 1999 ▸), and GSA is then isomerized to ALA by glutamate-1-semialdehyde-2,1-aminomutase (GSAM; EC 5.4.3.8; Ilag & Jahn, 1992 ▸). ALA formation is the rate-limiting step in tetrapyrrole biosynthesis (Tanaka & Tanaka, 2007 ▸).

**Figure 1:**
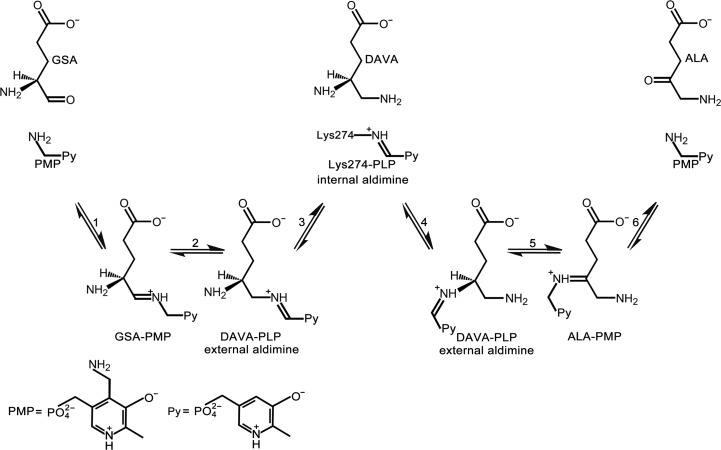
Schematic diagram for the reaction catalyzed by GSAM.

GSAM, also named glutamate-1-semialdehyde aminotransferase (GSA-AT), is a pyridoxamine 5′-phosphate (PMP)/pyridoxal 5′-phosphate (PLP)-dependent enzyme. Its topology corresponds to those of the other enzymes from subgroup II of the α-family of vitamin B_6_ enzymes (Mehta & Christen, 1994 ▸; Schulze *et al.*, 2006 ▸). Almost all B_6_ cofactors, including PLP and PMP, depend on the pyridinium moiety to stabilize high-energy anionic intermediates during reaction (Agnihotri & Liu, 2001 ▸). GSAM catalyzes the transamination of GSA substrate to ALA product by an unusual intramolecular exchange of amino and oxo groups *via* the intermediate 4,5-diaminovalerate (DAVA). The reaction starts with imine formation between PMP and the aldehyde of GSA (Fig. 1 ▸, step 1). Next, the double bond of this imine shifts to yield an external aldimine between PLP and the 5-amino group of DAVA (Fig. 1 ▸, step 2). The intermediate DAVA is then produced accompanied by the formation of an internal aldimine between PLP and the active-site lysine side chain (Fig. 1 ▸, step 3). The remainder of the reaction is the reverse of the first half (Fig. 1 ▸, steps 4, 5 and 6). Overall, during the first half of the reaction PMP is converted to PLP, while PMP is regenerated in the second half of the reaction upon ALA formation (Hennig *et al.*, 1997 ▸; Stetefeld *et al.*, 2006 ▸).

In *Arabidopsis thaliana*, two homologous genes, *AtGSA1* (*AT5G63570*) and *AtGSA2* (*AT3G48730*), share 90% sequence identity. All previous studies have been focused on structures of GSAM from prokaryotic species; thus, the crystallographic study of *At*GSA1, a representative from a higher plant, may provide further insight into this enzyme. Here, we present the high-resolution structure of *At*GSA1 at 1.25 Å resolution. Similar to *Synechococcus* GSAM, *At*GSA1 also displays asymmetry in its structure, which supports the negative cooperativity between monomers of GSAM.

## 2. Materials and methods   

### 2.1. Expression, purification and crystallization   

The gene for *At*GSA1 (*AT5G63570*) lacking the plastid-targeting sequences was amplified by PCR from cDNA (obtained from RT-PCR of total *A. thaliana* RNA) using the following primers containing sequences corresponding to the *Tobacco etch virus* (TEV) protease recognition site (in italics) and restriction sites (BamHI and XhoI; underlined): sense primer, 5′-CCTGGATCC *GAAAACCTGTATTTTCAGGGC*GTCGACGAGAAGAAGAAAAGTT-3′; antisense primer, 5′-CCTTTCTCGAGCTAGATCCTACTCAGTACCCTCTCA-3′. The gene product was cloned into pET-28a(+) (Novagen) to generate the pET-28a(+)-His_6_-*At*GSA1 plasmid. *Escherichia coli* BL21(DE3) cells containing the recombinant plasmid were incubated at 37°C on a rotary shaker at 180 rev min^−1^ until an OD_600_ of 0.8 was reached. The recombinant His_6_-tagged *At*GSA1 was expressed by induction with 0.4 m*M* IPTG at 16°C for 16 h. *E. coli* BL21(DE3) cells were lysed by sonication in buffer *A* (20 m*M* Tris–HCl pH 7.5, 200 m*M* NaCl) on ice. The His_6_-tagged protein was purified using a nickel–nitrilotriacetic acid column (Qiagen) and eluted in buffer *B* (buffer *A* supplemented with 200 m*M* imidazole). The His_6_ tag was cleaved by TEV protease at 4°C followed by size-exclusion chromatography in buffer *A* using a HiLoad 16/600 Superdex 200 pg column (GE Healthcare). The purified protein was concentrated by ultrafiltration in buffer *A*, flash-frozen in liquid nitrogen and stored at −80°C. For crystallization, the purified protein was diluted to a concentration of 8 mg ml^−1^. Crystals of *At*GSA1 were obtained using the sitting-drop vapour-diffusion method at 4°C in a drop consisting of 1 µl protein sample and an equal volume of well solution [0.15 *M* potassium bromide, 30%(*w*/*v*) PEG 2000 MME] taken from a 200 µl reservoir. Crystals suitable for X-ray data collection were optimized by the seeding method.

### 2.2. Data collection and structure determination   

The harvested crystals were cryoprotected stepwise in crystallization solution supplemented with 10 and 20%(*v*/*v*) glycerol and were then flash-cooled in liquid nitrogen. X-ray diffraction data were collected on beamline BL17U of Shanghai Synchrotron Radiation Facility at a wavelength of 0.979 Å at 100 K. The data were indexed, integrated and scaled using *DENZO* and *SCALEPACK* as implemented in *HKL*-2000 (Otwinowski & Minor, 1997 ▸). The structure of *At*GSA1 was solved by molecular replacement using the *Synechococcus* GSAM structure (PDB entry 2gsa; Hennig *et al.*, 1997 ▸) as the search model. Automatic model building was performed using *ARP*/*wARP* (Perrakis *et al.*, 1999 ▸), and manual model correction was performed in *Coot* (Emsley *et al.*, 2010 ▸). The model was further refined in *PHENIX* (Adams *et al.*, 2010 ▸) and the overall quality of the final structural model was assessed by *PROCHECK* (Laskowski *et al.*, 1993 ▸). Data-collection and structure-refinement statistics are summarized in Table 1 ▸. The coordinates and structure factors have been deposited in the Protein Data Bank with accession code 5hdm. Figures showing the protein structure were prepared using *PyMOL* (Schrödinger).

### 2.3. Spectral analysis   

Absorption spectra of purified *At*GSA1 were obtained with a UV-2550 spectrophotometer (Shimadzu) at room temperature. The scanning wavelength ranged from 250 to 750 nm. Spectra were corrected for buffer contribution.

### 2.4. Multiple sequence alignment   

*BLAST* searches were carried out on the NCBI website (http://blast.ncbi.nlm.nih.gov/Blast.cgi). Sequence alignment of GSAM from different species was performed using *Clustal Omega* at http://www.ebi.ac.uk/Tools/msa/clustalo/. The secondary-structure depiction was generated by *ESPript* (Robert & Gouet, 2014 ▸).

## 3. Results   

### 3.1. Overall structure   

**Figure 2:**
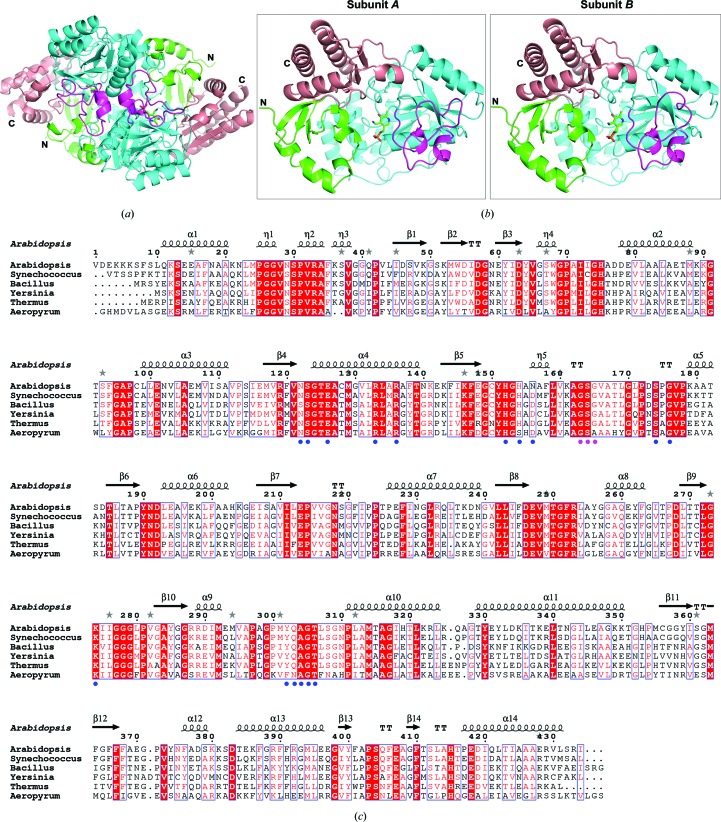
Overall structural analysis of *At*GSA1. (*a*) Stereoview of dimeric *At*GSA1 in cartoon representation with cofactors depicted in stick representation. The N-terminal domain, cofactor-binding domain and C-terminal domain are shown in green, cyan and salmon, respectively. The gating-loop region (residues 151–184) is shown in magenta. (*b*) Comparison of subunit *A* and subunit *B*. (*c*) Multiple sequence alignment of GSAM from *A. thaliana* (*At*GSA1, sequence without transit peptide), *Synechococcus elongatus*, *B. subtilis*, *Y. pestis*, *T. thermophilus* and *Aeropyrum pernix*. The secondary structure of *At*GSA1 is displayed above the sequences. Identical amino acids are in white on a red background. The similar residues are in red and boxed. Dots indicate gaps introduced during alignment. Blue circles denote the residues involved in negative cooperativity. Magenta circles denote the residues involved in gating-loop reorientation.

*At*GSA1 forms a dimer in the asymmetric unit. A size-exclusion chromatograpy study also indicated a dimeric state of *At*GSA1 in solution (data not shown). The mature *At*GSA1 protein (without the putative N-terminal chloroplast transit peptide of 40 residues) consists of 434 residues. Clear electron density in the structure of *At*GSA1 allowed the modelling of 428 residues in each monomer, with the first six N-terminal residues missing. The overall structure of *At*GSA1 is similar to other known GSAM architectures and consists of three sequentially arranged domains (Fig. 2 ▸): the N-terminal domain (Val1–Asp63, mature protein) comprises one α-helix and a three-stranded antiparallel β-sheet, the PMP/PLP-binding domain (Tyr64–Gly328), which is also the catalytic domain, contains a central seven-stranded β-sheet with one antiparallel and six parallel β-strands, and the C-terminal domain (Thr329–Ile434) is composed of a three-stranded antiparallel β-sheet with four helices covering the outer surface.

### 3.2. The asymmetry of *At*GSA1 in cofactor binding   

**Figure 3:**
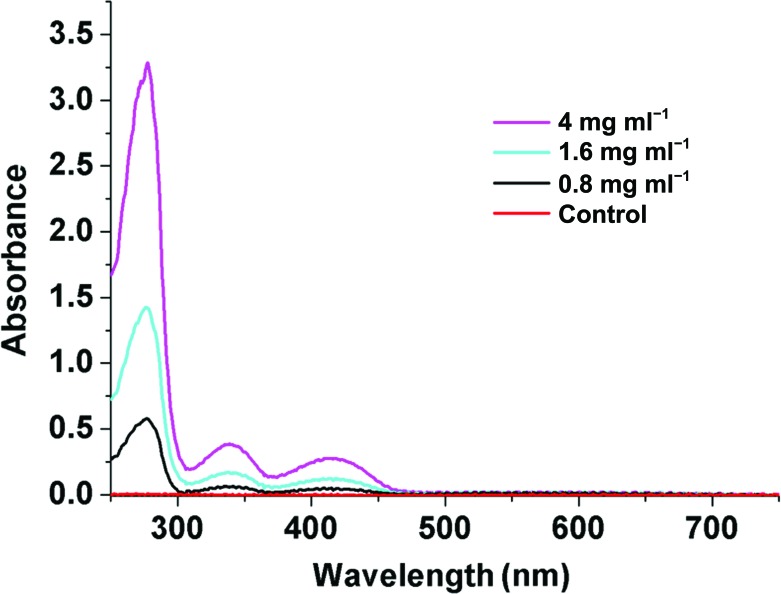
Absorption spectra of purified *At*GSA1. The enzyme was at different concentrations (0.8, 1.6 and 4 mg ml^−1^) in buffer consisting of 20 m*M* Tris–HCl pH 7.5, 200 m*M* NaCl. The buffer was used as a control.

Absorption spectral analysis of recombinant *At*GSA1 in solution indicates that the enzyme still retains the cofactors after purification in the absence of added cofactors. The enzyme has an absorption spectrum with a maximum at 338 nm and a relatively lower peak at 418 nm attributable to absorption by PMP and PLP, respectively (Fig. 3 ▸). This is consistent with the previous result that the enzyme in solution invariably contains both forms, unless preparations of GSAM are deliberately converted into either the double-PMP or the double-PLP form (Brody *et al.*, 1995 ▸; Pugh *et al.*, 1992 ▸; Smith *et al.*, 1991 ▸).

**Figure 4:**
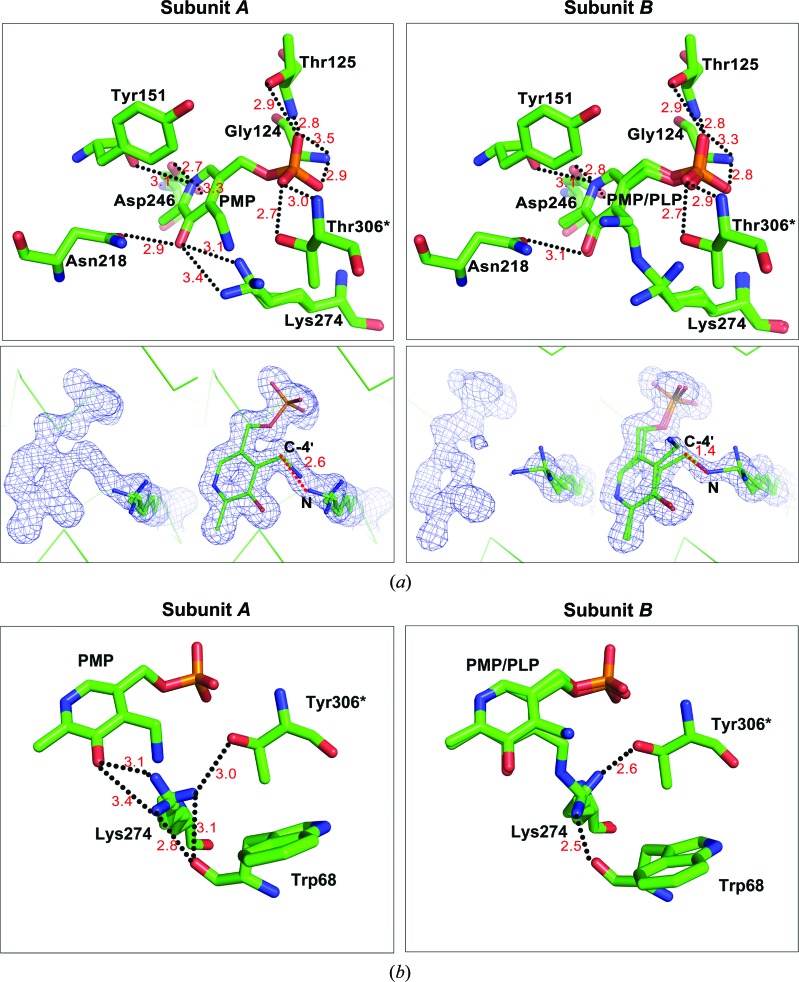
Close-up view of the cofactor-binding sites. (*a*) Residues interacting with the cofactor. The corresponding 2*F*_o_ − *F*_c_ electron-density maps of the cofactor and Lys274 are shown and contoured at 1.0σ. The cofactor in subunit *A* is PMP. The cofactor in subunit *B* is a mixture of PMP and PLP. Lys274 has multiple conformations in each monomer. (*b*) Interactions between Lys274 and the cofactor, Trp68 and Tyr306*. Hydrogen bonds are depicted as black dotted lines. Distances between the N atom of Lys274 and the C-4′ atom of the cofactor are depicted as red dotted lines. Distances in Å are displayed in red. The asterisk indicates the residue from the neighbouring subunit.

In agreement with the results of spectral analysis, the *At*GSA1 structure displays asymmetry in cofactor binding (Fig. 4 ▸). In the OMIT map of subunit *A* there is continuous electron density between the cofactor and Lys274. However, when PLP is modelled in the ligand density, the distance (2.6 Å) is not short enough to form a Schiff-base linkage between Lys274 and the cofactor (between the N atom of the ∊-amino group of Lys274 and the C-4′ atom of the cofactor), demonstrating that the cofactor in subunit *A* is PMP (Fig. 4 ▸ *a*). However, the PMP orientation is different from that previously observed in the PMP-containing subunit of *Synechococcus* GSAM or aspartate aminotransferase, in which the PMP cofactor is usually tilted by 20–30°, moving the amino group away from the catalytic lysine (Hennig *et al.*, 1997 ▸; Jansonius & Vincent, 1987 ▸; Stetefeld *et al.*, 2006 ▸). Instead, the orientation of PMP in subunit *A* is similar to that of PLP, as reported previously, with the amino group pointing towards the side chain of the active-site lysine (Fig. 4 ▸; Hennig *et al.*, 1997 ▸; Stetefeld *et al.*, 2006 ▸). Thus, the continuous electron density between PMP and Lys274 may be owing to the amino group of PMP and the side chain of Lys274 (in one of its multiple conformations) pointing towards each other. The PMP is recognized *via* hydrogen bonds to Gly124, Thr125, Tyr151, Asn218, Asp246 and Thr306* (the asterisk indicates a residue from the neighbouring subunit; Fig. 4 ▸ *a*).

In subunit *B*, both PMP and PLP are observed within the active site. In the OMIT map of subunit *B*, electron density between the cofactor and Lys274 is discontinuous. However, when PMP is modelled continuous electron density emerges and the distance (1.4 Å) is appropriate for covalent-bond formation between the cofactor and Lys274. Therefore, both PMP and PLP are modelled in the ligand density with occupancies of 0.54 and 0.46, respectively. The amino group of PMP points away from Lys274 and PLP forms a Schiff-base linkage with the ∊-amino group of Lys274 (Fig. 4 ▸ *a*), similar to that previously reported in the *Synechococcus* GSAM structure (Hennig *et al.*, 1997 ▸; Stetefeld *et al.*, 2006 ▸). The side chain of Lys274 has three conformations in each subunit: (i) interacting with Trp68 and Thr306*, (ii) interacting with PMP by hydrogen bonds in the PMP form and (iii) covalently binding to the cofactor in the PLP form (Fig. 4 ▸ *b*). Except for Lys274, the residues involved in cofactor fixation in subunit *B* are similar to those in subunit *A* (Fig. 4 ▸ *a*).

### 3.3. The asymmetry of *At*GSA1 in the gating-loop conformation   

**Figure 5:**
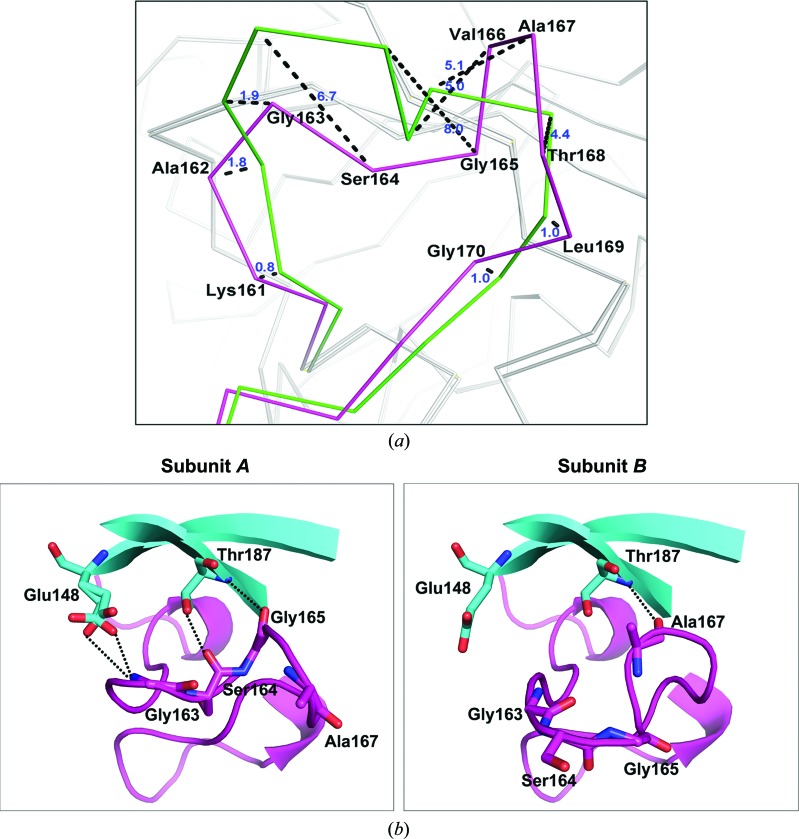
Conformations of the gating loop. (*a*) Superposition of the gating loops of subunit *A* (magenta) and subunit *B* (green) in ribbon representation. C^α^ deviations of Lys161–Gly170 are depicted as black dashed lines. Deviation values in Å are shown in blue. (*b*) The difference in hydrogen-bond interactions between subunit *A* and subunit *B*. Hydrogen bonds are depicted as dotted lines.

**Figure 6:**
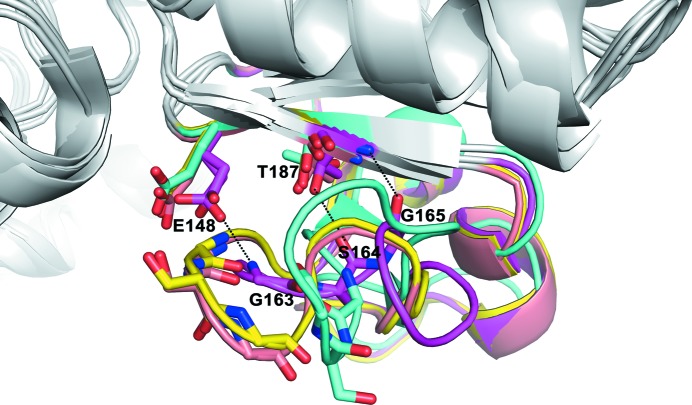
Comparison of gating-loop regions from different GSAM structures. The gating loops from subunit *A* of *At*GSA1 (PDB entry 5hdm), *B. subtilis* GSAM (PDB entry 3bs8) and *Synechococcus* GSAM in the double-PMP form (PDB entry 2hoz) and the PMP/PLP form (PDB enyry 2hp2) are shown in magenta, cyan, yellow and salmon, respectively. Conserved residues corresponding to Gly163, Ser164, Gly165, Glu148 and Thr187 from *At*GSA1 are indicated by single-letter residue codes. Hydrogen bonds involved in gating-loop fixation are depicted as dotted lines.

Different conformations of the gating loop can be correlated with the states of the cofactor and the corresponding catalytic intermediate in the active site. Superposition of subunits *A* and *B* of *At*GSA1 shows asymmetry reflecting the mobility of the gating-loop region (residues 151–184; Fig. 5 ▸ *a*), which has been shown to control access to the active site and limit the dissociation of the DAVA intermediate (Stetefeld *et al.*, 2006 ▸). In subunit *A*, three hydrogen-bond interactions are found to fix the gating loop and keep it in the open state, which are between Gly163 and Glu148, between Ser164 and Thr187 and between Gly165 and Thr187 (Fig. 5 ▸ *b*). By comparing the gating loop of subunit *A* with the corresponding region in all of the previously described GSAM structures, we found that this characteristic of gating-loop fixation has not previously been observed (Fig. 6 ▸). As shown in the *At*GSA1 structure, subunit *A* only binds PMP and the gating loop is fixed in the open state, consistent with previous reports that the catalytic reaction is initiated by PMP (Stetefeld *et al.*, 2006 ▸). As the orientation of PMP in subunit *A* is similar to that of PLP in subunit *B* (Fig. 4 ▸), it is possible that subunit *A* of *At*GSA1 is in the state (Fig. 1 ▸, the end of step 6) where PMP has just been regenerated in order to restart the reaction.

**Figure 7:**
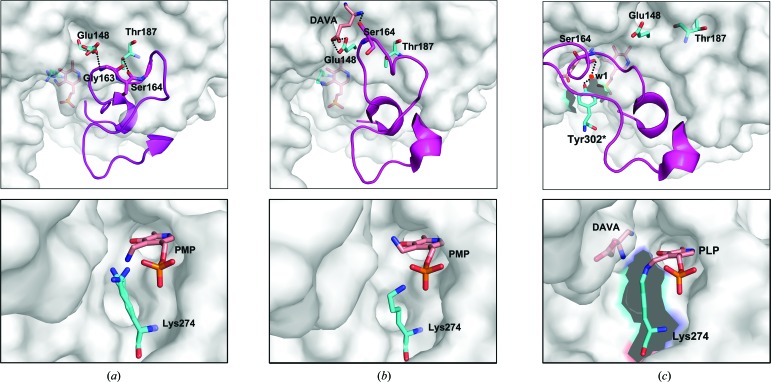
A proposed model of tthe gating-loop transition between the open, ready-to-close and closed conformations (upper panel) and the corresponding active site (lower panel). (*a*) The gating loop is fixed in the open state by hydrogen-bond interactions between Glu148 and Gly163 and between Thr187 and Ser164. PMP with the amino group pointing towards Lys274 has just been regenerated to restart the reaction. (*b*) The substrate (DAVA as the substrate analogue) interacts with Glu148 and Ser164 to interrupt the hydrogen-bond network between the gating loop and residues Glu148 and Thr187. Thus, the gating loop is released and ready to close. The PMP cofactor is tilted by 20–30°, with the amino group moving away from the catalytic lysine. (*c*) The gating loop moves to cover the active-site pocket during the catalytic process and Tyr302* forms a water-mediated hydrogen bond to Ser164. PMP is converted to PLP by forming a Schiff-base linkage to the lysine side chain. The asterisk indicates the residue from the neighbouring subunit.

Compared with subunit *A*, the gating loop of subunit *B* undergoes a dramatic conformational change as demonstrated by the large C^α^ deviations of the residues Lys161–Gly170. The maximum deviation of 8.0 Å occurs at Gly165, followed by Ser164 (6.7 Å), Ala167 (5.1 Å), Val166 (5.0 Å) and Thr168 (4.4 Å) (Fig. 5 ▸ *a*). The overall (root-mean-square deviation) r.m.s.d. value of C^α^ atoms for the superposition of subunits *A* and *B* is 0.35 Å. In addition, two forms of cofactor are observed within the active site of subunit *B*. Thus, the gating loop of subunit *B* may be in an intermediate state, and the disrupted network of hydrogen bonds between Gly163, Ser164 and Gly165, and Glu148 and Thr187 may result in the gating loop of subunit *B* becoming ready to close. Our data reveal the mobility of the gating-loop residues Gly163, Ser164 and Gly165, which are important for the reorientation of the gating loop. Previous studies have shown that Ser164 can interact in some respects with the DAVA molecule (substrate analogue) in the double-PMP-form GSAM structure (PDB entry 2hoz) with the gating loop in the open state and that Ser164 also contributes significantly to the helical conformation of the closed gating loop by forming water-mediated hydrogen bonds to Tyr302* and catalytic intermediates (Stetefeld *et al.*, 2006 ▸). In addition, the importance of Ser164 has been revealed by site-directed mutagenesis (Bishop *et al.*, 1999 ▸). Thus, we propose a model based on the *At*GSA1 structure and the *Synechococcus* GSAM structure (PDB entries 2hoz and 2hp2; Fig. 7 ▸). Hydrogen-bond interactions between Gly163 and Ser164 and Glu148 and Thr187 keep the gating loop in the open state to allow the entry of substrate (Fig. 7 ▸ *a*); next, the substrate interacts with Ser164 and Glu148 to release the gating loop, accompanied by large C^α^ deviations of Lys161–Gly170, and the gating loop then becomes ready to close (Fig. 7 ▸ *b*); finally, the gating loop covers the active-site pocket during the catalytic process and Tyr302* forms a water-mediated hydrogen bond to Ser164 (Fig. 7 ▸ *c*).

## 4. Discussion   

Hennig and coworkers demonstrated the asymmetry of dimeric *Synechococcus* GSAM both in the crystal structure (PDB entry 2gsa) and in solution, and accordingly speculated on a negative-cooperativity mechanism of GSAM (Hennig *et al.*, 1997 ▸). Negative cooperativity describes a phenomenon in multi-subunit proteins where the binding of the first ligand induces a conformational change in the protein so that the binding of subsequent ligands becomes more difficult (Conway & Koshland, 1968 ▸; Levitzki & Koshland, 1969 ▸). The evidence supporting such a cooperative catalytic mechanism in GSAM is as follows. Firstly, through crystallographic studies, several asymmetric *Synechococcus* GSAM structures have been reported and hydrogen-bond-mediated intersubunit crosstalk has been proposed (Hennig *et al.*, 1997 ▸; Stetefeld *et al.*, 2006 ▸). Besides, the *Arabidopsis* GluTR dimer is also asymmetric (Zhao *et al.*, 2014 ▸). Since a model of the complex of GluTR and GSAM has been proposed (Moser *et al.*, 2001 ▸), GSAM and GluTR could possibly behave asymmetrically during catalysis in a coordinated way. Secondly, GSAM shows biphasic kinetic behaviour in solution (Hennig *et al.*, 1997 ▸) and invariably contains a mixture of PMP and PLP unless preparations of GSAM are deliberately converted into either the double-PMP or the double-PLP form (Brody *et al.*, 1995 ▸; Pugh *et al.*, 1992 ▸; Smith *et al.*, 1991 ▸). Besides, the asymmetry of the gating-loop conformation in solution has been proved (Campanini *et al.*, 2013 ▸). However, the negative-cooperativity theory has also been challenged by some symmetric structures as both monomers can obviously adopt the same state simultaneously. The crystal structure of *Bacillus subtilis* GSAM (PDB entry 3bs8) shows structural symmetry, including the gating-loop region in the open state, as well as identical cofactor (PMP) binding in each monomer (Ge *et al.*, 2010 ▸). GSAM structures from *Thermus thermophilus* (PDB entry 2e7u; RIKEN Structural Genomics/Proteomics Initiative, unpublished work), *Aeropyrum pernix* (PDB entry 2zsl; RIKEN Structural Genomics/Proteomics Initiative, unpublished work) and *Yersinia pestis* (PDB entry 4e77; Center for Structural Genomics of Infectious Diseases, unpublished work) are also symmetric. However, in our study, *At*GSA1 displays asymmetry in cofactor binding as well as in the gating-loop conformation. Our results support the negative-cooperativity mechanism of GSAM. According to the alignment results, *At*GSA1 shares 73, 58, 54, 53 and 43% sequence identity with GSAM_Syn_ from the cyanobacterium *Synechococcus*, GSAM_Bsu_ from *B. subtilis*, GSAM_Ype_ from *Y. pestis*, GSAM_Tth_ from *T. thermophilus* and GSAM_Ape_ from *A. pernix*, respectively. Structure superposition resulted in r.m.s.d. values of 0.629 Å for *At*GSA1 and GSAM_Syn_, 0.976 Å for *At*GSA1 and GSAM_Ype_, 0.986 Å for *At*GSA1 and GSAM_Tth_, 1.013 Å for *At*GSA1 and GSAM_Bsu_ and 1.203 Å for *At*GSA1 and GSAM_Ape_ on C^α^ atoms. A phylogenetic analysis revealed that *At*GSA1 is closely evolutionally related to GSAM_Syn_ from the cyanobacterium *Synechococcus* (Supplementary Fig. S1). Therefore, the negative cooperativity of *At*GSA1 could have evolved from cyanobacterial GSAM through endosymbiotic biogenesis of the chloroplast.

Allosteric communication in proteins is characterized by evolutionarily conserved structural networks of amino-acid interactions (Lockless & Ranganathan, 1999 ▸; Süel *et al.*, 2003 ▸). Based on the structural analysis of *At*GSA1 and the intersubunit communication theory (Stetefeld *et al.*, 2006 ▸), we found that both the interface helix (Asn122–Thr139; Stetefeld *et al.*, 2006 ▸) and the interface loop (Tyr302–Thr306) are involved in electrostatic crossover interactions transmitting signals of active-site occupancy and gating-loop state to the neighbouring subunit (Supplementary Fig. S2). All of the residues involved in negative cooperativity are conserved (Fig. 2 ▸ *c*). Through the network of interactions, GSAM exhibits negative cooperativity between monomers in a coordinated way. According to Stetefeld and coworkers, the monomers of the GSAM dimer exist in two complementary conformations and switch between open and closed forms (Stetefeld *et al.*, 2006 ▸), demonstrating the most extreme form of negative cooperativity, which corresponds to ‘half-of-the-sites reactivity’ (Koshland, 1996 ▸). However, it remains elusive why GSAM shows negative cooperativity. The possible reasons could be as follows. Firstly, the kinetic behaviour of the enzyme with both subunits in the PLP form reveals a significantly decreased GSA turnover (Tyacke *et al.*, 1993 ▸). Thus, negative cooperativity of GSAM would prevent the enzyme being converted into the almost inactive double-PLP form during normal activity (Hennig *et al.*, 1997 ▸). Secondly, enzymes involved in the tetrapyrrole-biosynthesis pathway have been proposed to be organized in multiprotein complexes, in which the assembly of cooperating proteins is coordinated to direct the transfer of metabolic intermediates from one enzyme to the next (Wang & Grimm, 2015 ▸). In addition, a complex between GluTR and GSAM has been proposed (Moser *et al.*, 2001 ▸). Thus, GSAM and GluTR could possibly exhibit negative cooperativity in a coordinated way to increase the catalytic efficiency.

## Supplementary Material

PDB reference: *At*GSA1, 5hdm

Supporting Information: Supplementary Figures S1 and S2.. DOI: 10.1107/S2053230X16007263/us5093sup1.pdf

## Figures and Tables

**Table 1:** Data-collection and structure-refinement statistics for *At*GSA1 Values in parentheses are for the highest resolution shell.

Data collection	
Space group	*P*2_1_2_1_2_1_
Unit-cell parameters (Å, °)	*a* = 64.1, *b* = 109.3, *c* = 115.5, α = β = γ = 90.0
Wavelength (Å)	0.9793
Resolution (Å)	50.00–1.25 (1.29–1.25)
No. of unique reflections	224024
Completeness (%)	95.0 (96.0)
Multiplicity	3.9 (3.7)
〈*I*/σ(*I*)〉	22.1 (3.9)
*R*_merge_ or *R*_sym_ †	0.050 (0.320)
Refinement statistics	
Resolution (Å)	28.88–1.25
No. of measured reflections	204630
*R*_work_/*R*_free_ ‡	0.126/0.150
No. of atoms	
Protein	6700
Ligand	47
Water	1091
Average *B* factor (Å^2^)	
Protein	15.83
Ligand	18.45
Water	33.51
R.m.s.d., bond lengths (Å)	0.007
R.m.s.d., bond angles (°)	1.175
Ramachandran plot	
Favoured (%)	98.12
Allowed (%)	1.66
Outliers (%)	0.22
*R*_p.i.m._	0.026
*R*_meas_	0.057
CC_1/2_	0.916

† *R*_merge_ = 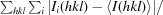 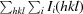, where *I*_*i*_(*hkl*) is the observed intensity and 〈*I*(*hkl*) is the average intensity obtained from multiple observations of symmetry-related reflections after rejections.

‡ *R*_work_ = 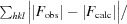 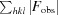, where *F*_obs_ and *F*_calc_ are the observed and calculated structure factors, respectively. *R*_free_ = 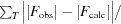 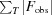, where *T* is a test data set of 5% of the reflections which were omitted during refinement.
