# Supplementary material for: Crystal structure of glutamate-1-semialdehyde-2,1-aminomutase from Arabidopsis thaliana
Source: Acta Crystallogr F Struct Biol Commun. 2016 May 23;72(Pt 6):448–56. doi: 10.1107/S2053230X16007263 (PMC4909244; doi:10.1107/S2053230X16007263)
Supplement: Supplementary file 1 [file f-72-00448-sup1.pdf]

STRUCTURAL BIOLOGY
COMMUNICATIONS

Volume 72 (2016)

Supporting information for article:

Crystal structure of glutamate-1-semialdehyde-2,1-aminomutase from *Arabidopsis thaliana*

Yingxian Song, Hua Pu, Tian Jiang, Lixin Zhang and Min Ouyang

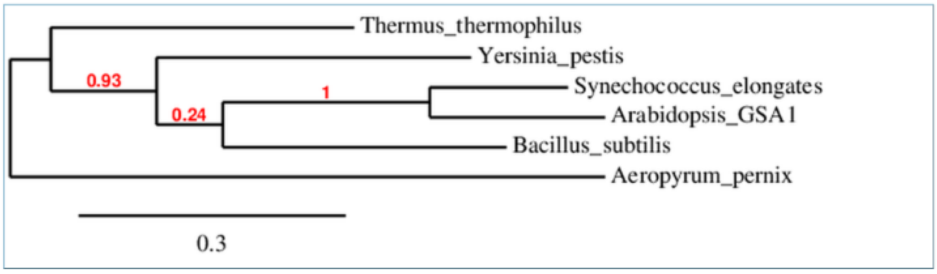

Figure S1 Phylogenetic relationship of GSAM from *Arabidopsis thaliana*, *Synechococcus elongates*, *Bacillus subtilis*, *Yersinia pestis*, *Thermus thermophiles*, and *Aeropyrum pernix*. Bootstrap analyses were computed with 1,000 replicates, and the values of percentage are shown on the branches.

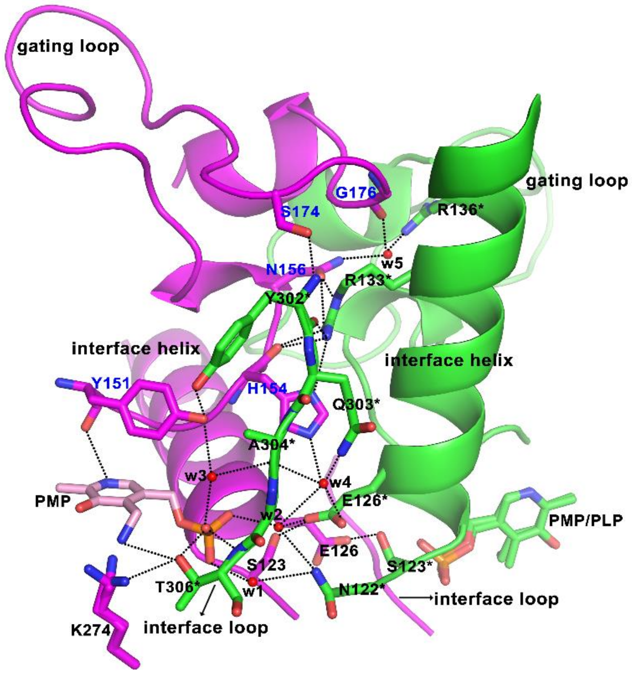

Figure S2 A proposed model displaying hydrogen-bond network involved in the negative cooperativity between monomers of GSAM. Specifically, (i) cofactor and Lys274 send active-site information by interacting with the neighboring interface helix/loop through the following hydrogen bonds, cofactor-w1-Asn122*, cofactor-w2-Glu126*, cofactor-w3-Ala304*, cofactor/Lys274-Thr306*. (ii) Gating-loop (residues 151-184) information is transmitted through interactions with the neighboring interface helix/loop, i.e. Tyr151-Tyr302*, His154-w4-Glu126*/Gln303*/Ala304*, His154-Arg133*, Asn156-Arg133*, Asn156-w5-Arg136*, Ser174-Tyr302*, Gly176-w5-Arg136*. (iii) Interface helixes from the two monomers can interact

with each other (Ser123-Glu126*, Glu126-Ser123*). (iv) Besides, hydrogen-bond interactions also exist between cofactor and gating loop (cofactor-Tyr151), between interface helix and interface loop from the same monomer (Tyr302*-Arg133*, Gln303*/Ala304*-w4-Glu126*), between interface loop and residues participating in substrate pocket lining (Ser30-Gly305*, Arg33-Thr306*) (data not shown). Subunit A and B of *At*GSA1 is shown in magenta and green, respectively. All water molecules (red spheres) shown are conserved in at least *Synechococcus* GSAM and *At*GSA1 structures. Hydrogen-bond interactions are depicted as dotted dashes. The * indicates the residue from the neighboring subunit.
